# Downregulation of Cyclophilin A by siRNA diminishes non-small cell lung cancer cell growth and metastasis via the regulation of matrix metallopeptidase 9

**DOI:** 10.1186/1471-2407-12-442

**Published:** 2012-10-02

**Authors:** Zhe Qian, Xiaoting Zhao, Mei Jiang, Wenyun Jia, Chunyan Zhang, Yue Wang, Baolan Li, Wentao Yue

**Affiliations:** 1 Department of Cellular & Molecular Biology, Beijing TB and thoracic tumor research Institution/ Beijing Chest Hospital, Capital Medical University, 97 Beimachang, Tongzhou, Beijing, China; 2 General medicine Department, Beijing Chest Hospital, Capital Medical University, 97 Beimachang, Tongzhou, Beijing, China

**Keywords:** Cyclophilin A, Non-small cell lung cancer, Proliferation, Metastasis, Matrix metallopeptidase 9

## Abstract

**Background:**

Cyclophilin A (CypA) is a cytosolic protein possessing peptidyl-prolyl isomerase activity that was recently reported to be overexpressed in several cancers. Here, we explored the biology and molecular mechanism of CypA in non-small cell lung cancer (NSCLC).

**Methods:**

The expression of CypA in human NSCLC cell lines was detected by real-time reverse transcription PCR. The RNA interference-mediated knockdown of CypA was established in two NSCLC cell lines (95C and A549). 239836 CypA inhibitor was also used to suppress CypA activity. Tumorigenesis was assessed based on cellular proliferation, colony formation assays, and anchorage-independent growth assays; metastasis was assessed based on wound healing and transwell assays.

**Results:**

Suppression of CypA expression inhibited the cell growth and colony formation of A549 and 95C cells. CypA knockdown resulted in the inhibition of cell motility and invasion. Significantly, we show for the first time that CypA increased NSCLC cell invasion by regulating the activity of secreted matrix metallopeptidase 9 (MMP9). Likewise, suppression of CypA with 239836 CypA inhibitor decreased cell proliferation and MMP9 activity.

**Conclusions:**

The suppression of CypA expression was correlated with decreased NSCLC cell tumorigenesis and metastasis.

## Background

Lung cancer, the leading cause of cancer death worldwide, can be divided into two types: non-small cell lung cancer (NSCLC) and small cell lung cancer (SCLC). NSCLC accounts for approximately 85% of lung cancers. Despite much research, progress in the diagnosis and treatment of NSCLC remains limited; the five-year survival rate is only 15% [1]. Nevertheless, treatment at an early stage, especially during the precancerous stage, could increase the five-year survival rate by three- to four-fold. Apart from conventional screening methods such as imaging and pathology, the molecular diagnosis of NSCLC is becoming increasingly important [2]. Targeted therapy has found a place in the treatment of NSCLC in recent years as it is highly tailored, more effective, and has fewer side-effects (1). Increased understanding of the mechanism of NSCLC will enable us to exert a crucial influence on carcinogenesis. So far, however, the molecular mechanism underlying the genesis of NSCLC remains unclear.

Cyclophilins were originally identified as cell-binding proteins of the immunosuppressive drug, Cyclosporin A. The founding member of cyclophilins is cyclophilin A (CypA), an 18-kDa cytosolic protein that is ubiquitously expressed in prokaryotes and eukaryotes [3,4]. CypA is also known as peptidyl-prolyl isomerase (PPI) A, as it specifically catalyzes the cis-trans isomerization of peptidyl-prolyl bonds. Thus, CypA plays important roles in protein folding, assembly, and trafficking, as well as immunoregulation and cell signaling [5-8]. CypA is implicated in several diseases, including viral infection, cardiovascular disease, inflammatory diseases, and cancer [9-12].

Though CypA was discovered in the past century, and despite the fact that its overexpression was first demonstrated in hepatocellular carcinoma in 1998 [13], the role of CypA in cancer has until recently drawn insufficient attention. Various cancers, including lung cancer, colorectal cancer, pancreatic cancer, breast cancer, squamous cell carcinoma, and melanoma exhibit upregulated CypA [14-22]. Some researchers have investigated the function of CypA during tumor progression, including the stimulation of proliferation, blockade of apoptosis, regulation of metastasis, and malignant transformation. The discussion of CypA in lung cancer began in 2003 when Campa reported that the level of CypA protein in lung cancer specimens was seven-fold higher than that in adjacent non-diseased lung tissues [23]. CypA was subsequently reported to promote proliferation and metabolism, and to restrain apoptosis in NSCLC cells [15]. Similar results were obtained in SCLC [16]. Even so, the function of CypA in lung cancer remains incompletely understood.

The objective of this study was to determine the effect of the CypA suppression on NSCLC cell growth and metastasis *in vitro*, and to gain insight into the relevance of CypA to NSCLC biology and its underlying mechanisms. As expected, CypA expression was higher in lung cancer cells and enhanced cell growth by stimulating proliferation, colony formation, tumorigenesis, and metastasis by stimulating cell migration and invasion. Furthermore, we detected the secreted matrix metalloproteinase 2 (MMP2) and MMP9, which are correlated with metastasis in NSCLC, and we found that CypA enhanced the activity of secreted MMP9.

## Methods

### Cell culture

Three human lung adenocarcinoma cell lines, A549, A2, and H1299; two human large cell lung carcinoma cell lines, 95C and H460; and one small cell lung cancer cell line, H446, were cultured in RPMI 1640 medium (Invitrogen, Carlsbad, CA, USA) supplemented with 10% fetal bovine serum (FBS; Gibco, Los Angeles, CA, USA). Human embryo lung fibroblasts (MRC5) were maintained in DMEM supplemented with 10% FBS. The cells were maintained at 37°C in a humidified chamber containing 5% CO_2_ and 95% air.

### CypA RNAi lentivirus generation

Four CypA-targeting oligonucleotides serving as RNAi candidates were designed based on the full-length human CypA cDNA sequence and cloned into the pGCsi-H1/Neo/GFP vector (Shanghai Genechem Co. Ltd., Shanghai, China). CypA-Si2 (CTGACTGTGGACAACTCGAAT), which matches the sequence located at nucleotides 559–579 of the CypA cDNA, proved to be the most effective at decreasing the CypA mRNA level and was used to knock down endogenous CypA in the following experiments. A nonsilencing-siRNA (NS-siRNA, TTCTCCGAACGTGTCACGT) was used as a negative control. Oligonucleotides encoding CypA-Si2 or NS-siRNA together with a loop separating the complementary sequences were synthesized and inserted into the pGCL-GFP lentivirus construct, which contained an H1 promoter and an ampicillin resistant cassette (Shanghai Genechem Co. Ltd.). The recombinant virus was packaged using a Lentivector Expression System (Shanghai Genechem Co. Ltd.), according to the manufacturer’s instructions.

### Recombinant lentiviral particle infection of target cells

Target cells were plated into 96-well culture plates at 5,000/well. Cells were infected with recombinant virus carrying CypA-siRNA or NS-siRNA 24 h later. GFP expression was detected via fluorescence microscopy (Nikon, Tokyo, Japan) to determine the infection efficiency. Cells were cultured for an additional 2 weeks prior to harvest, at which time CypA expression was assessed by quantitative real-time PCR (qRT-PCR) and Western blot analysis.

### Reverse transcription PCR and qRT-PCR analyses

Total RNA was extracted using TRIzol Reagent (Invitrogen) with the RNA quality being assessed by formaldehyde-agarose gel electrophoresis. First-strand cDNA was obtained by reverse-transcription using Moloney murine leukemia virus reverse transcriptase (Promega, Madison, WI, USA) as instructed by the manufacturer. qRT-PCR was performed using Power SYBR Green PCR Master Mix (Applied Biosystems, Foster City, CA, USA) in a total volume of 25 μL with 2 μL of cDNA, and detected using an ABI7500 Real-Time PCR System (Applied Biosystems). The real-time PCR conditions for CypA and β-actin were: 95°C for 10 min, followed by 40 cycles of 95°C for 30 s, 56°C for 30 s, and 72°C for 30 s with the primers CypA sense (5’-CATACGGGTCCTGGCATCT-3’), CypA antisense (5’-TGCTGGTCTTGCCATTCC-3’), β-actin sense (5’-TTAGTTGCGTTACACCCTTTC-3’), and β-actin antisense (5’-GCTGTCACCTTCACCGTTC-3’). β-actin was used as internal loading controls. Relative mRNA levels are presented as 2^-ΔCT^. Three independent experiments were completed; each reaction was performed in triplicate. All data are shown as means ± SEM. A no-template control (NTC) was used to avoid genomic DNA contamination.

### Western blot analysis

Whole-cell lysates were harvested in ice-cold lysis buffer (10 mM Tris–HCl, pH 7.4, 1 mM EDTA, 0.1% Triton X-100, and 0.1% SDS) containing protease inhibitors (2 μg/mL aprotinin, 10 μg/mL antipain, 2 μg/mL pepstatin, and 2 mM benzamide). After the removal of cell debris by centrifugation (12,000 × *g*, 10 min), the protein concentration in the supernatants was measured using bicinchoninic acid protein assay reagent (Pierce Chemical Co., Rockford, IL, USA) according to the manufacturer’s instructions. Ten micrograms of total protein were subjected to SDS-PAGE and wet-transferred to nitrocellulose membranes. The membranes were probed with anti-human CypA polyclonal rabbit serum (Santa Cruz Biotechnology, Santa Cruz, CA, USA) at a 1:500 dilution and anti-GAPDH antibodies (CoWin Biotech, Shanghai, China) diluted 1:5,000 in 5% (w/v) nonfat dry milk in TBST (50 mM Tris–HCl, 138 mM NaCl, and 0.1% Tween-20, pH 7.6). Secondary antibodies conjugated to horseradish peroxidase (Santa Cruz Biotechnology) were diluted 1:5,000. Signals were detected using the SuperSignal West Femto Chemiluminescent Detection System (Pierce Chemical Co.) and exposed to Kodak X-OMAT film.

For the detection of ERK1/2, p38, JAK2, and STAT5, rabbit anti-ERK1/2 antibody (Abcam, Cambridge, England, UK), mouse anti-pERK antibody (Abcam, Cambridge, England, UK), rabbit anti-p38 antibody (Abcam, Cambridge, England, UK), mouse anti-pp38 antibody (Abcam, Cambridge, England, UK), mouse anti-JAK2 antibody (Abcam, Cambridge, England, UK), rabbit anti-pJAK2 antibody (Abcam, Cambridge, England, UK), rabbit anti-STAT5 antibody (Abcam, Cambridge, England, UK), and mouse anti-pSTAT5 antibody (Abcam, Cambridge, England, UK) were used.

### High-content cell cycle analysis

The Cellomics ArrayScan HCS Reader was used to quantify cell parameters by immunofluorescence staining. In brief, cells were cultured in 96-well plates at 37°C for 24hours, washed three times with phosphate-buffered saline, and then fixed in 4% paraformaldehyde for 10 min followed by 0.2% TritonX-100 for 15 min at room temperature. After blocking of non-specific binding sites in 3% BSA for 30 min, cells were incubated with anti-CypA primary antibodies overnight at −4°C and treated with anti-rabbit IgG-TRITC secondary antibody for 30 min at room temperature. The expression level of CypA protein was measured with the Cellomics ArrayScan HCS Reader using the ArrayScanTM software.

### Cell proliferation assay

Tumor cells (3,000/well) were seeded in flat-bottom 96-well plates. The next day, the cells were serum-starved for 24 h and exposed to 0.2% BSA. Cell proliferation was evaluated by a 3-(4, 5-dimethyl-thiazol-2yl)-5-(3-carboxymethoxyphenyl)-2-(4-sulfophenyl)-2H-tetrazolium (MTS; Promega) assay, which was performed at a fixed time every day for the next 5 days. A total of 20 μL of MTS was added to each well, followed by incubation for 3 h. The absorbance was recorded at 490 nm with an EL-800 universal microplate reader (Bio-Tek Instruments, Winooski, VT, USA). This assay was repeated three times in triplicate. Cell doubling-time was calculated using doubling time online calculator [24].

### Colony formation assay

Three-hundred cells were suspended in 2 mL of culture medium and seeded in 6-well plates. The cells were maintained for 10 days with a change of media every 3 to 4 days. The number of colonies with >50 cells in each well was counted on the 10th day. The colonies were visualized and counted by the trypan blue exclusion method. The assay was repeated three times in triplicate.

### Anchorage-independent growth assay

A total of 1,000 cells were resuspended in 1 mL of 0.6% agarose in 6-well plates coated with a 1.2% agarose bed. Triplicate cultures of each cell type were maintained for 21 days; the medium was changed every 7 days. The number of colonies >50 μm (~100 cells) in diameter per well was counted manually with the aid of Alpha View Analysis Tools (Alpha Innotech Corp., San Leandro, CA, USA). All experiments were performed in triplicate and repeated three times.

### Cell migration assay

Cell motility was assessed by two assays. For the wound healing assay, confluent cell monolayers were wounded with a sterile pipette tip and cultured in serum-free medium in 6-well plates. The wounds were observed at 0, 12, and 24 h along the scratch, and representative images of fixed positions were acquired with a phase-contrast microscope. The wound areas were measured using Alpha View Analysis Tools, and the percentage wound closure was determined.

A migration assay was performed in a 24-well Transwell unit containing an 8-μm pore size polycarbonate membrane (Costar, Cambridge, NY, USA) as reported previously [25]. After starvation for 12 h, the cells were suspended and plated in the upper compartment with serum-free medium. The lower compartment was filled with medium containing 10% FBS for use as a chemoattractant. After 24 h,the cells in the upper compartment were removed completely by gentle swabbing. Cells migrating to the lower surface of the membrane were determined using crystal violet. The number of cells on the lower surface of the membrane was counted in five microscopic fields at 200× magnification. Triplicate samples were tested. The data are presented as means ± SEM.

### Cell invasion assay

The invasion assay was determined by transwell chamber as reported previously [25]. Briefly, cells were starved for 12 h, suspended, and seeded in the up-per compartment on Matrigel Matrix (5 μg/mL; BD Pharmingen, San Diego, CA, USA)-coated 24-well Transwell units (Costar). RPMI 1640 medium supplemented with 10% FBS was added to the lower compartment for use as a chemoattractant. After incubation for 24h and 48 h respectively, cells attached to the lower surface of the membrane were stained by crystal violet. The number of cells on the lower surface of the membrane was counted in five microscopic fields at 400× magnification. Triplicate samples were assayed. The data are presented as means ± SEM.

### Gelatin zymography

Gelatinolytic activity and quantity in conditioned media were analyzed by gelatin zymography. In brief, serum-free conditioned medium was centrifuged to remove cellular debris and then subjected to non-reducing SDS-PAGE using an 8% separating gel containing 0.1% gelatin. Subsequently, the gels were washed twice in 2.5% Triton X-100 for 30 min at room temperature to remove SDS and incubated in reaction buffer (50 mM Tris, 0.2 M NaCl, and 5 mM CaCl_2_) for 48 h at 37°C to hydrolyze the copolymerized protein substrate in a zone around their electrophoresed position. The gels were subsequently stained with 0.5% Coomassie brilliant blue R-250 to visualize the digested areas as clear bands against a blue background of undegraded gelatin. The gels were then scanned and analyzed using Alpha View Analysis Tools.

### CypA inhibitor 239836 function studies

Cell proliferation assay and gelatinolytic activity was also assessed in the presence of CypA inhibitor 239836(Merck, Darmstadt, Germany). In brief, A549 and 95C cells were seeded onto 96-well plates or 6-well plates. 239836 (0, 0.1, 1, and 10 μg/mL) was added 24 hours later. The vehicle (DMSO, 0.3 μl/ml) was used as a control. Cell growth curve and the activity of MMP9 were detected.

### Statistical analysis

The data are presented as the means ± SEM of at least three independent experiments. Statistical analysis was performed using Student’s *t*-test. Unless otherwise indicated, *P*<0.05 was deemed significant.

## Results

### CypA expression in NSCLC cell lines

**Figure 1:**
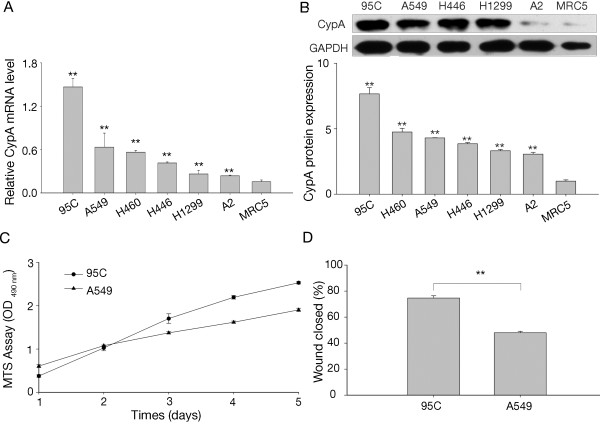
**Expression of CypA in six lung cancer cell lines and MRC5 cells. A**, CypA mRNA expression in five NSCLC cell lines (95C, A549, H460, H1299, and A2), one SCLC cell line (H446) and MRC5. The 95C, A549, H460, H446, H1299, and A2 cells exhibited 12.02-, 5.19-, 4.62-, 3.40-, 2.28-, and 1.94-fold increases, respectively, in CypA mRNA expression compared to MRC5 cells. The relative mRNA level is presented as 2^-ΔCT^. All data shown are means ± SEM from three separate experiments. **P<0.01 (compared with MRC5, *t*-test). **B**, CypA protein level in 95C, A549, H1299, A2, H446 and MRC5 cell lines were detected by western-blot, GAPDH was used as internal loading controls (upper panel). CypA protein level was also measured by immunofluorescence staining. The fluorescence intensity was quantified by Cellomics ArrayScan HCS Reader using the ArrayScanTM software (lower panel). The CypA protein level normalized to MRC-5. **P<0.01 (compared with MRC5, *t*-test) **C**, Proliferation of 95C and A549 cells. OD_490_ values were determined daily at predetermined time points. The doubling-time of 95C and A549 cells was 24h and 43 h respectively. The data are expressed as the mean ± SEM of triplicate values from three separate experiments. **D**, Mobility of 95C and A549 cells. The percent distance of wound closure at 24 h following wound generation was calculated from three separate areas and is expressed as the mean±SEM. **P<0.01 (*t*-test).

To examine CypA expression in lung cancer cells, we tested the CypA mRNA level in six human lung cancer cell lines, together with one normal human embryo lung fibroblast line (MRC5) as an NTC. The mRNA levels were quantified by real-time PCR using specifically designed primers for CypA; β-actin was used as an internal normalizer to avoid variation. Compared to the MRC5 cells, all lung cancer cells expressed significantly higher levels of CypA mRNA, particularly 95C cells (Figure 1A). The level of CypA protein was detected by western-blot analysis. As shown in Figure 1B (upper panel), CypA protein was highly expressed in lung cancer cells except A2 cell line. We also used high-content cell cycle analysis to determine the CypA protein level, similar result was obtained as shown in Figure 1B. To determine the potential function of CypA in lung cancer cell pathophysiology, we investigated the proliferative and migratory capacities of 95C and A549 cells. Our data suggest that 95C cells had enhanced cell growth and metastasis compared to A549 cells (Figure 1C and D), which inspired us to further investigate the correlation between CypA and lung cancer cell growth and metastasis.

### Cell proliferation was suppressed by the inhibition of CypA levels

**Figure 2:**
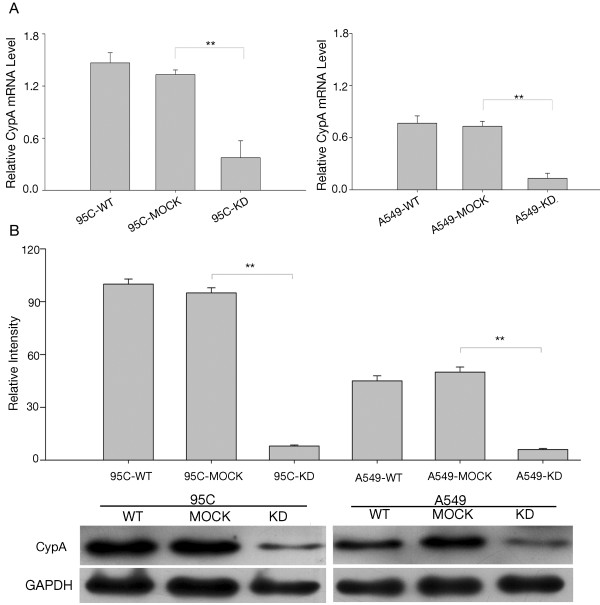
**Interference with CypA expression in 95C and A549 cells. A**, CypA mRNA expression in 95C and A549 cells with CypA-targeting RNAi. The relative mRNA level is presented as 2^-ΔCT^. The data are the means ± SEM of three separate experiments. **B**, CypA protein levels in 95C and A549 cells with CypA-targeting RNAi. Cell lysates prepared from each group were screened using Western blotting with anti-CypA antibodies. The data are expressed as the mean ± SEM of triplicate values from three separate experiments. **P<0.01 (*t*-test).

**Figure 3:**
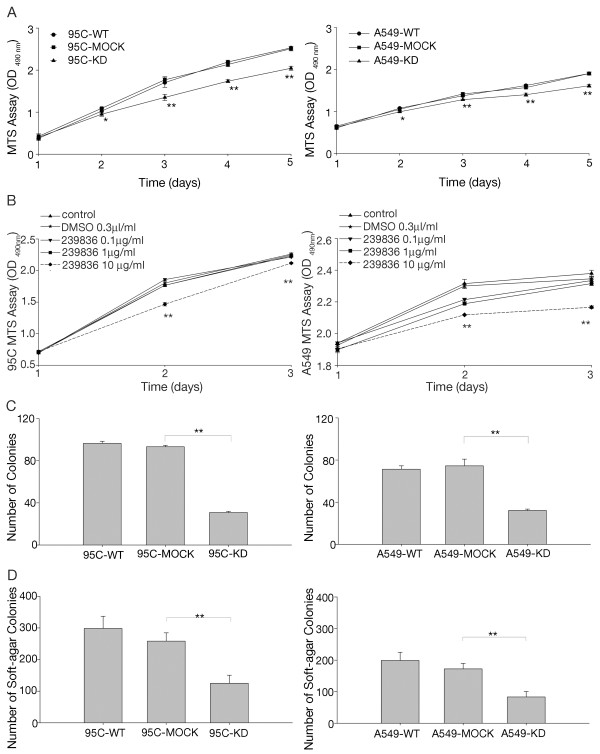
**Effect of CypA on tumorigenesis. A**, CypA depletion inhibited the proliferation of 95C and A549 cells. OD_490_ values were determined daily at predetermined time points. The data are expressed as means ± SEM of triplicate values from three separate experiments. *P<0.05, **P<0.01 (*t*-test). 95C, 95C-MOCK, 95C-KD cell doubling-time was 24h, 26h, 31h respectively. And cell doubling-time of A549, A549-MOCK, A549-KD was 43h, 44h, 49h respectively. B, the effect of 239836 on cell proliferation was detected by MTS assay. The data are expressed as means ± SEM. **P<0.01 (*t*-test) **C**, CypA-assisted 95C and A549 cells avoided density-dependent growth inhibition. Colony formation after 10 days was quantified by counting the number of colonies per well. The values represent the mean number of colonies±SEM from three separate wells. **P<0.01 (*t*-test). **D**, The knockdown of CypA suppressed the anchorage-independent proliferation of 95C and A549 cells. Colony formation after 21 days was quantified by counting the number of colonies per well. The values represent the mean number of colonies (the mean±SEM) from three separate wells. **P<0.01 (*t*-test).

A lentivirus-based RNAi delivery system with short-interfering RNAs against CypA was used to infect 95C and A549 cells to determine the function of CypA (referred to as 95C-KD and A549-KD). A nonsilencing sequence was used as a negative control (referred to as 95C-MOCK and A549-MOCK). 95C-KD and A549-KD showed an approximately 80% reduction in CypA mRNA; moreover, CypA protein expression was knocked down by 90% compared to the parental cells (referred to as 95C-WT and A549-WT) (Figure 2A and B). Cells treated with the nonsilencing sequence showed no difference in CypA (Figure 2A and B). As indicated in Figure 3A, the KD cells exhibited decreased growth beginning on the second day. Up to day 5, proliferation of the KD cells was dramatically slower than that of the WT and MOCK cells. These data indicate that CypA was depleted effectively and might be a key factor stimulating proliferation in NSCLC cells.

To determine whether CypA plays a role in cell proliferation, A549 and 95C cells were incubated with CypA inhibitor 239836, at concentrations of 0, 0.1, 1, and 10 μg/mL for 48 hours. MTS assay was performed. As shown in Figure 3B, the proliferation of A549 and 95C was inhibited by the treatment of 239836 in a dose-dependent manner.

### The suppression of CypA inhibits NSCLC cell tumorigenesis

To gain additional insight into the effect of CypA on NSCLC growth, cell tumorigenesis was assessed by colony formation and anchorage-independent growth assays. The former indicated that the colony-forming efficiency of KD cells was less than half that of WT and MOCK cells after 10 days (Figure 3C). Thus, CypA may overcome the density-dependent inhibition of growth in NSCLC cells. Similarly, in an anchorage-independent growth assay, the number of WT colonies was nearly twice that of KD colonies, while there were no differences between WT and MOCK cells (Figure 3D). Our data demonstrate that the suppression of CypA resulted in marked inhibition of soft-agar colony formation.

### CypA expression increase cell proliferation by up-regulation of MAP kinase pathway

**Figure 4:**
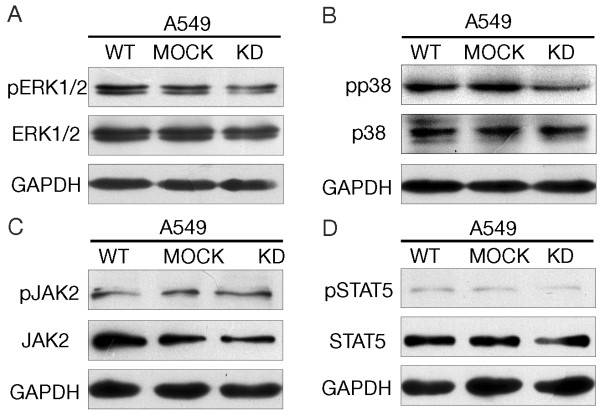
**The phosphorylation level of ERK1/2 and p38 was deregulated by the suppression of CypA expression. **Western-blot analysis of A549, A549 MOCK, A549 KD with antibodies to ERK1/2 (**A**), p38(**B**), JAK2(**C**), STAT5(**D**) and their phosphorylated forms were carried out. GAPDH was used as internal loading controls.

To explore how the CypA modulates cell proliferation signaling pathways, some central regulatory molecules of MAP kinase and JAK2 pathways were examined using western blot analysis. The phosphorylation levels of both ERK1/2 and p38 were decreased in A549 KD cells (Figure 4A and B). We also analyzed the phosphorylation levels of JAK2 and STAT5 in these cells, but no obvious change was observed (Figure 4C and D). Thus, CypA appears to be involved in the MAPK kinase signal pathway (ERK1/2 and p38).

### CypA suppression decreases NSCLC cell metastasis

**Figure 5:**
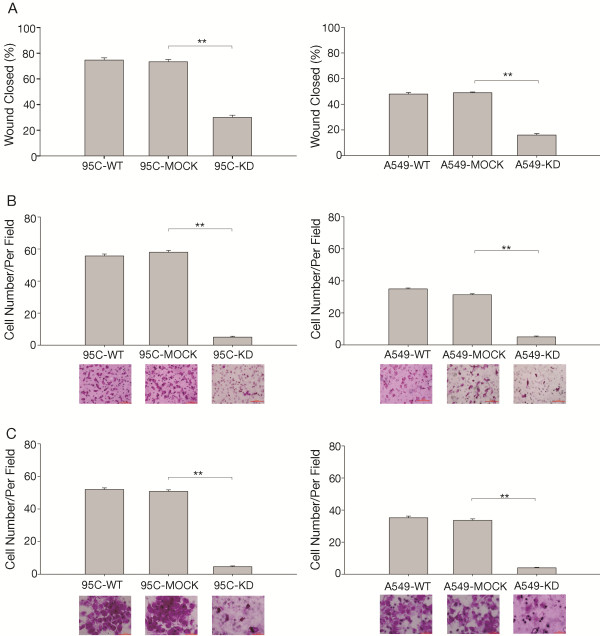
**The rates of migration and invasion in CypA interfered 95C and A549 cells with A, Migration was investigated using a wound healing assay. **The percent areas of wound closure at 24 h following wound generation were calculated from three separate areas and are expressed as the mean±SEM. **P<0.01 (*t*-test). Photomicrographs were taken after 24 h (original magnification, ×100). **B**, Other migration assays were performed using a Transwell unit. Cells on the underside of the insert filters were fixed, stained, and counted under a microscope. The data are expressed as means ± SEM of triplicate values from three separate experiments. **P<0.01 (*t*-test). Photomicrographs were taken after 24 h (original magnification, ×200). **C**, The *in vitro* invasive properties of 95C and A549 cells was tested using a Matrigel-coated Transwell unit. Cells on the underside of the insert filters were fixed, stained, and counted under a microscope. The data are expressed as means ± SEM of triplicate values from three separate experiments. **P<0.01 (*t*-test). Photomicrographs were taken after 48 h (original magnification, ×400).

Metastasis is an important characteristic of malignant cancer cells. To further assess the influence of CypA on 95C and A549 cell metastasis, we investigated its effect on cell migration and invasion. In the invasion assay, none of the cells were observed when cells were incubated for 24h, and we count the number of cells on the lower surface of the membrane when cells were incubated for 48h. Both cell migration, as determined using wounding healing and Transwell assays (Figure 5A and B), and cell invasion (Figure 5C) were inhibited in KD cells compared to the corresponding controls. These findings indicate that CypA could promote NSCLC cell metastasis.

### CypA inhibition correlates with the down-regulation of MMP9 activity

**Figure 6:**
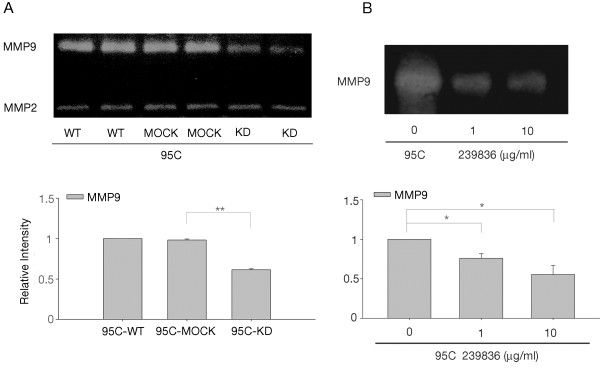
**Zymographic analysis of MMP9 and MMP2 activity in 95C cells. A**, Conditioned media prepared from each cell group were screened using gelatin zymographic analysis. The densities of MMP9 were determined and plotted. The data are expressed as the means ± SEM of triplicate values from three separate experiments. **P<0.01(*t*-test). **B**, Conditioned media prepared from CypA inhibitor 239836 treated cells as well as control group were screened using gelatin zymographic analysis. The gels were scanned and analyzed using Alpha View Analysis Tools. *P<0.05.

A series of mechanisms are involved in the metastasis of NSCLC, and MMPs play particularly critical roles [26]. Two key MMPs, MMP2 and MMP9, were differently influenced by CypA in NSCLC cells, as detected by gelatin zymography (Figure 6A). MMP9 activity in KD cells was decreased, while that in WT and MOCK cells was similar (Figure 6A). However, no significant differences were detected among WT, MOCK, and KD cells in terms of MMP2 activity (data not shown). In order to check the change of MMP9 activity was resulted from suppression of CypA, the CypA inhibitor 239836 was used. 95C cells were incubated with CypA inhibitor 239836 for 48 hours and MMP9 activity was detected. As shown in Figure 6B, MMP9 activity in 239836 treated cells were significantly decreased, showing a dose-dependent manner. In summary, our findings suggest that CypA stimulates cell proliferation and might promote metastasis by upregulating the activity of MMP9 in NSCLC, without changing MMP2 activity.

## Discussion

Targeted therapy represents a tremendous leap forward in cancer treatment. The mutation of epidermal growth factor receptor is a highlight of lung cancer diagnosis and therapy [27]. Therefore, elucidating the mechanisms of novel molecular targets may contribute to improving lung cancer treatment. Previous reports have demonstrated that CypA is overexpressed in cancers such as lung, pancreatic, colorectal, and breast cancer. CypA overexpression was considered to play important roles in cancer pathogenesis and act like a "molecular switch" [28], since it is known to regulate signaling via prolyl isomerization. Nonetheless, the mechanism underlying the effect of CypA on the biological behavior of lung cancer cells has not been fully elucidated.

In this study, we showed that the knockdown of CypA in human NSCLC cells inhibited cell proliferation, increased sensitivity to density-dependent inhibition, and down-regulated anchorage-independent cell growth. This is in agreement with a previous report showing that CypA is a key promoter of tumor cell growth and tumorigenesis [15]. Notably, CypA knockdown dramatically inhibited cell migration and invasion by NSCLC cells, suggesting that CypA has a significant impact on the metastasis of NSCLC cells. Furthermore, we investigated the mechanism of action of CypA in NSCLC cells, and detected enhanced MMP9 activity. To our knowledge, this study for the first time correlates CypA with metastasis and MMP9 in NSCLC cells. Our data indicate that CypA plays a crucial role in the proliferation, motility, and invasionof NSCLC cells.

The expression of CypA in lung cancer tissue was approximately seven-fold higher than that in adjacent nonmalignant tissue [14]. Herein, we showed that compared to MRC5 cells, CypA expression was higher in several lung cancer cell lines, including five NSCLC (95C, A549, H460, A2, and H1299) and one SCLC (H446) cell lines. Interestingly, proliferation and wound healing assays indicated that 95C had a greater proliferative and migratory capacity than A549, suggesting that elevated CypA expression in NSCLC cells might influence cell growth and metastasis.

It has been thought that CypA accelerates cell growth by stimulating cell proliferation, tumorigenesis, and metabolism, and by inhibiting apoptosis [15]. Our proliferation and tumorigenesis data are consistent with those of previous reports, but the mechanism by which CypA acts on cell growth remains unclear. We also checked whether cell apoptosis was regulated by CypA and cell apoptosis was not affected by the suppression of CypA expression (data not shown). Recent studies have pointed out that PPI activity is required for CypA-induced cell proliferation, and that several growth-related signaling molecules, including ERK1/2, Jak2, p38, and Stat5 [10,16,20], are stimulated by CypA in cancer cells. In present research, some important regulatory molecules of MAP kinase and JAK2 signaling pathways were determined. Our data indicate that CypA enhanced cell growth by up-regulating MAPK kinase pathway (ERK1/2 and p38) in NSCLC cells. But JAK2/STAT5 was not involved in the CypA regulating pathway.

Metastasis is the primary cause of morbidity and mortality in cancer patients. Besides its role in cell growth, the involvement of CypA in metastasis has also been investigated. Stable CypA RNA-interfered breast cancer and osteosarcoma cells showed reduced migratory capacity [20,29]. CypA is also involved in the attraction and migration of monocytes or vascular smooth muscle cells in rheumatoid arthritis and cardiovascular disease by irritating adhesion molecules and regulating MMP9 secretion [10,30]. These reports inspired us to explore the effect of - CypA on metastasis in NSCLC cells. KD cells exhibited a deficiency in migratory capacity compared with WT and MOCK cells. Invasion is another characteristic of metastasis. To some extent, invasive ability is even more important because the first step in metastasis involves passing through a basement membrane (BM), which is the major physical obstacle to cancer cell metastasis [31]. Our invasion assay suggested that KD cells could not pass through the Matrigel Matrix, which is similar to the BM; however, WT and MOCK cells could. Nevertheless, the mechanism of CypA in metastasis remains a mystery. It is thought that CypA might disrupt the F-actin structure in osteosarcoma cells or the regulation of JAK2 signaling in breast cancer cells and metastatic melanoma cell lines [19,22]. Thus far, no studies have focused on NSCLC cells. In our study, the phosphorylation level of JAK2 was not changed when CypA expression was inhibited.

We hypothesized that a relationship exists between CypA and MMPs, in particular MMP2 and MMP9, as CypA stimulates MMP expression via the ligand CD147 [27,32,33] . The suppression of CD147 in breast cancer cells inhibited MMP2 and MMP9 production and cell invasion *in vitro*[34], while the invasive properties conferred on inflammatory cells by CypA are a result of MMP9 stimulation [35]. We suppressed CypA in NSCLC cells without changing CD147 expression (data not shown). Gelatin zymography showed that MMP9 was down-regulated in CypA supressed cells compared to control cells, while the expression of MMP2 did not change significantly. This is consistent with a previous report showing that CypA increased MMP9, but not MMP2, expression via CD147 in rheumatoid arthritis [29]. Therefore, CypA may promote metastasis by upregulating MMP9 activity in NSCLC cells.

The initial step of tumor cell invasion is characterized by BM breakdown, a process dependent on type IV collagen-degrading enzymes, mainly MMP2 and MMP9 [36]. MMPs are a family of proteases that are required for the invasion of tumor cells into surrounding connective tissues, intravasation and extravasation from blood vessels, and metastasis to distant organs [37]. MMP2 and MMP9, otherwise known as gelatinases, are strongly upregulated in cancers of the lung, colon, breast, skin, and prostate, which are correlated with enhanced tumor invasiveness and metastasis [38]. MMP9 digests decorin; elastin; fibrillin; laminin; types IV, V, XI, and XVI collagen; and gelatin. Among these, laminin serves as an important component of the BM. MMP9 can also activate growth factors, such as proTGF-β and proTNF-α [39]. The inhibition of MMP9 reduced the number of colonies formed in the lung of mice [40]. As demonstrated in this study, CypA upregulated the activity of MMP9 in NSCLC cells, which could help elucidate the mechanism of CypA effect on lung cancer metastasis. Furthermore, MMP9 is widely accepted as a prognostic marker in NSCLC; its expression level is correlated with survival [41]. Whether CypA could be used as a prognostic marker in NSCLC was evaluated by Howard using tissue microarray immunohistochemistry; however, the results were negative [14]. Further research into CypA may change this using larger samples or alternate methods.

## Conclusions

We demonstrated for the first time that CypA knockdown decreased metastatic activity in NSCLC cells, and that strong CypA expression could activate MAPK signaling pathway and plays an important role in the cell proliferation. Therefore, CypA is a potential indicator of tumor prognosis. In addition, we speculate that CypA regulates the activity of MMP9, a key factor in tumor metastasis, although this should be investigated further *in vivo*, and the CypA-centered regulatory network should be elucidated in detail. Thus, CypA shows promise as a novel therapeutic target and/or prognostic indicator for lung cancer.

## Abbreviations

NSCLC: Non-small cell lung cancer; SCLC: Small cell lung cancer; CypA: Cyclophilin A3; MMP: Matrix metallopeptidase; NTC: No-template control.

## Competing interests

The authors promised there were not any actual or potential conflicts of interest in this research.

## Authors’ contributions

WY designed the study and helped to draft the manuscript. ZQ and XZ performed the study and drafted the manuscript. ZQ, XZ, MJ and JW carried out the experiments and interpreted the experimental findings. CZ participated in the cell culture. YW develop conditions for the RT-PCR experiments. BL and ZQ performed the statistical analysis. “All authors read and approved the final manuscript.”

## Pre-publication history

The pre-publication history for this paper can be accessed here:

http://www.biomedcentral.com/1471-2407/12/442/prepub
